# Edge Machine Learning for AI-Enabled IoT Devices: A Review

**DOI:** 10.3390/s20092533

**Published:** 2020-04-29

**Authors:** Massimo Merenda, Carlo Porcaro, Demetrio Iero

**Affiliations:** 1 Department of Information Engineering, Infrastructure and Sustainable Energy (DIIES), University Mediterranea of Reggio Calabria, 89124 Reggio Calabria, Italy; porcarocarlo@libero.it (C.P.); demetrio.iero@unirc.it (D.I.); 2 HWA srl-Spin Off dell’Università Mediterranea di Reggio Calabria, Via Reggio Campi II tr. 135, 89126 Reggio Calabria, Italy

**Keywords:** artificial intelligence, machine learning, Internet of Things, edge devices, deep learning

## Abstract

In a few years, the world will be populated by billions of connected devices that will be placed in our homes, cities, vehicles, and industries. Devices with limited resources will interact with the surrounding environment and users. Many of these devices will be based on machine learning models to decode meaning and behavior behind sensors’ data, to implement accurate predictions and make decisions. The bottleneck will be the high level of connected things that could congest the network. Hence, the need to incorporate intelligence on end devices using machine learning algorithms. Deploying machine learning on such edge devices improves the network congestion by allowing computations to be performed close to the data sources. The aim of this work is to provide a review of the main techniques that guarantee the execution of machine learning models on hardware with low performances in the Internet of Things paradigm, paving the way to the Internet of Conscious Things. In this work, a detailed review on models, architecture, and requirements on solutions that implement edge machine learning on Internet of Things devices is presented, with the main goal to define the state of the art and envisioning development requirements. Furthermore, an example of edge machine learning implementation on a microcontroller will be provided, commonly regarded as the machine learning “Hello World”.

## 1. Introduction

The Internet of Things (IoT) scenario [1,2] has gained a lot of notoriety in recent years. It encompasses an infrastructure of software and hardware that connects the physical world with the Internet. Due to the explosive growth of interest in this paradigm, the number of IoT devices has increased dramatically in recent years. It has been estimated that by 2025, more than 75 billion devices will be connected to the Internet [3], leading to an economic impact on the global market. IoT devices typically have limited computing power, small memories, and could generate large amounts of data. Low-power and connected systems including mainly sensors will be used in our homes, cities, vehicles, and industries. Cloud computing might be suitable for the IoT sector growth but the delay caused by the data transfer is unacceptable for some tasks (e.g., health monitoring), in addition to possible bandwidth saturation. So, due to the greater numbers of connected devices, the only-cloud processing could become impractical and would lead to greater latency, bandwidth decrease, and privacy and reliability problems [4]. Hence the need to bring the calculation as locally as possible, incorporating intelligence on end devices to limit cloud traffic. This means giving a sort of “consciousness” to the devices that become able to interact also in the absence of the connection, elaborating complex behavior, and adapting to rapidly changing situations, a sort of “Internet of Conscious Things”. Unfortunately, limitations in the computational capabilities of resource-scarce devices restrict the implementation of complex machine learning (ML) algorithms on them, although several frameworks based on software agents provide reliable and effective solutions for the optimizations of different edge computing implementation [5,6,7]. The tasks that can be delivered to the edge elements are related to low-data fusion [8], while for a deeper understanding of the data (e.g., decision-making purposes) it is necessary to assign the calculation to more efficient systems. However, the transferring of raw data to cloud servers increases communication costs, causes delayed system response, and exposes private data. To address these issues, a practical solution is to consider processing data closer to its sources and transmitting to remote servers only the data needed for further cloud processing. Edge computing refers to computations being performed as close to data sources as possible, instead of remote locations.

**Figure 1:**
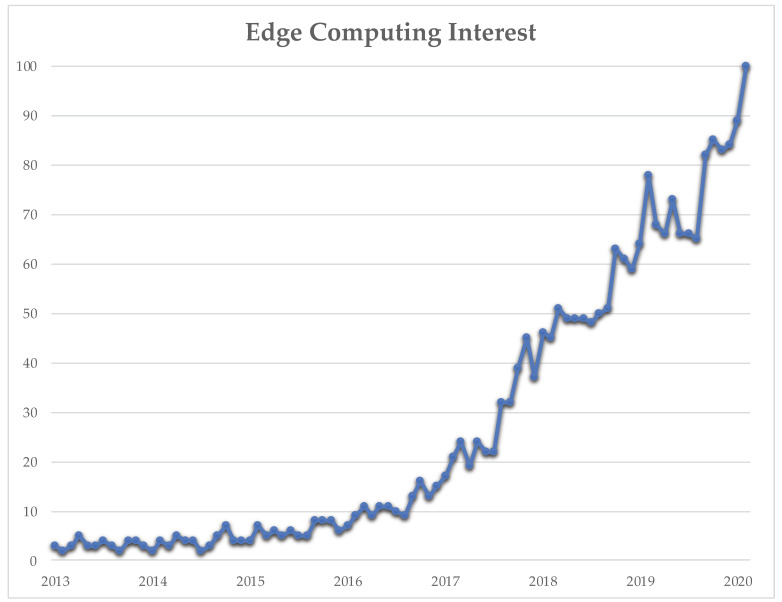
Edge computing interest (Google Trends).

Both search engines and libraries were used to write the review. As reported on Google Trends [9], in these years there has been an increase of interest from the scientific community on the topic and issues of *edge computing* (Figure 1) and a total of 6342 papers are reported from Scopus [10]. The keywords employed to obtain the papers to be analyzed was *edge computing*.

In order to discern which papers to use, only papers written in English were considered. Furthermore, to observe the recent evolution in this field, the selected papers were published from the years 2014 to the first months of 2020. Finally, a total of about 100 papers were utilized to compile this review.

The distribution of the papers regarding the country of the first author providing the countries that investigate edge computing systems are mainly China, USA, UK, and Italy. China is the country with the highest number of papers with a total of 2193 papers, 34.7% of the total.

The solution paved from the usage of edge machine learning (eML) is a viable way to meet the latency, scalability, and privacy challenges described earlier.

In order to make possible this scenario, efficient artificial intelligence (AI) algorithm could be deployed on the devices as SVM (support vector machine) [11,12], deep learning (DL) [13,14] and, *inter alia,* neural networks (NNs) [15,16]. It is noteworthy that NNs require less computational power in the application phase than in the training phase. This feature can be exploited to execute algorithms of AI on devices with limited resources, as microcontrollers (MCUs), allowing local data processing. In fact, in the learning phase, a large amount of data is used to calculate the weights and biases of the network, thus requiring a performing machine in this phase. Once the learning phase has been completed and the network has been trained, the model can be used for inference (in statistic field, it is the process of using data analysis to deduce properties of an underlying probability distribution) with a lower computational request. In fact, once the coefficients have been calculated, they are stored in the program memory and the AI algorithm can be executed on a device with a low capacity in terms of RAM (random access memory). ML algorithms are used for different topics such as smart cities [5,17,18,19,20,21,22], computer vision [23,24,25,26,27], health care [28,29,30,31,32,33], automotive [34,35,36,37], and others. Concerning these fields, there are different examples of how machine learning could be brought on edge devices. Anandhalli and Baligar in 2017 proposed in [18] a video processing algorithm that identified and counted the vehicles on a road. The algorithm was run on a Raspberry Pi3 (1.2 GHz quad-core ARMv8, 1 GB of RAM) with a built-in camera, using the library OpenCV5 (Open Source Computer Vision Library [38]). In [23], the authors developed a face recognition algorithm for law enforcement agencies within a smart city. A portable wireless camera mounted on the uniform of a police officer is used to capture the images that are then passed on to a Raspberry Pi3 to perform facial recognition. The Viola-Jones algorithm [39] is first used to identify faces in the images, then the ORB algorithm [40] extracts the peculiar features from the faces that are then transmitted to an SVM algorithm in the cloud to identify people. Therefore, for IoT purposes, the devices must be sufficiently powerful to perform certain tasks, even though, in general, it is possible to insert AI even in any embedded devices by exploiting a certain class of algorithms. The use of ML algorithms also allows the extension of the average battery life of the device, with power saving one of the fundamental tasks of the IoT world. For example, authors in [28] focused on increasing the battery life of a device used for e-health purposes by optimizing sampling times and data transfers using ML algorithms. Any unnecessary data that are transferred, stored, and processed appear to be a potential waste of energy. Using an SVM algorithm, based on the RBF (radial basis function) [41], kernel function, and varying the sampling frequency, it was possible to increase the life of the device from 2 weeks to years (997 days). In the same scenario considered, the wearable sensor platform for health-care in a residential environment (SPHERE) [42] is used to classify human activity into three categories: Sedentary, moderate, and sportive. The data measured by an accelerometer are sent to the MCU using Serial Peripheral Interface (SPI) protocol. After processing the data, they are packed by the microcontroller and sent using *advertisement mode* to a Bluetooth Low Energy (BLE) radio, which transmits data outside the smart home to a central unit. The use of SVM can also be found in [43], which highlights how the use of ML algorithms allows greater efficiency and low consumption in predicting patients’ seizures. In fact, crisis prediction is a difficult task due to the variability of the electroencephalography (EEG) signal depending on the patient. A neurostimulator able to identify and react to a principle of crisis can facilitate applications that would not otherwise be feasible, such as the possibility of generating a stimulus to suppress the crisis itself. The article demonstrates how ML techniques like SVM can be used to identify possible crises in each patient. The use of NN on embedded devices can be found in [44], which deals with the use of learning algorithms on an inexpensive robot built to perform sense-motor tasks. The robot, in particular, learns to trace objects by identifying the peculiarities of the object itself. The algorithm uses a CNN (convolutional neural network) to combine color, brightness, motion, and audio information while training is carried out using supervised ML algorithms. The images are properly analyzed to eliminate redundant input data. A Motorola board populated with a 68HC11, an 8-bit MCU with Complex Instruction Set Computer (CISC) architecture is used, which converts and processes the gyroscope outputs and generates sound feedback and timing signals for 14 servomotors that allow 3 degrees of freedom for each of the four legs and two degrees of freedom for the robot’s head. A Charge-Coupled Device (CCD) camera is mounted on the top of the robot, which acts as the robot’s eye.

As seen from the previous papers, ML algorithms can be implemented on devices with limited computational power and this can be used to improve the IoT field [45,46], thus enabling the *edge computing*. A related term, *fog computing*, describes an architecture where the cloud is extended to be closer to the IoT end devices, thereby improving latency and security by performing computations near the network edge [4]. Therefore, also for the fog computing, the task is to bring the processing phase closer to where the data are generated but the main difference is where the “intelligence” is located. In fog computing the processing phase is at the LAN (local-area network) level, in a fog node or IoT gateway. In edge computing, data are mainly processed directly on the devices to which the sensors are attached (physically very close to the sensors). The closer to the sensor, the better it is in terms of privacy and power consumption because of the reduction of the energy request related to data transmission. In this scenario, a variety of possibilities of energy harvesting from different sources paves the way for AI-enabled passive or semipassive IoT sensor platforms [47,48,49,50,51].

This survey focused on ML systems deployed on edge devices. Section 2 provides a comparison between the ML algorithms implementable in edge computing. In Section 3, the process of bringing ML to the edge is analyzed. Section 4 describes edge server-based architectures while in Section 5 the wireless standards for AI-enabled IoT devices are introduced. Section 6 provides edge-specific solutions for offloading techniques, detailing the differences of the joint computation alternatives. Section 7 deals with privacy issues and how to protect user privacy in uploading data. Section 8 describes the edge implementations of the training phase, in ML design. In Section 9, an example of edge machine learning implementation is provided, commonly regarded as the machine learning “Hello World”. The conclusions are, finally, drawn in Section 10.

## 2. Machine Learning Algorithms

We now discuss ML algorithms that could be used in resource-constrained settings at the edge of the network. The machine learning algorithms introduced in the next paragraphs are the most used in the papers that afford the problem of bringing AI in devices with resource-constrained hardware.

### 2.1. Deep Learning

**Figure 2:**
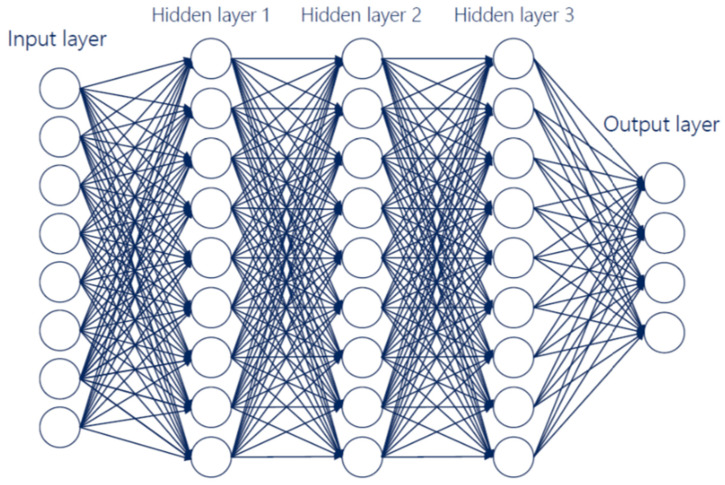
Deep Neural Network (DNN) example.

A deep learning model can be thought as a combination of weights and biases [52]. These parameters are varied by an optimization function (ADAM [53] optimization algorithm is generally used) based on an objective function (loss function or reward function, if the learning is, respectively, supervised or reinforcement) that measures the predictive power of the model. Following a training phase, the AI algorithm identifies an underlying pattern between the data, predicting a value as a function of the inputs’ data. Depending on the training phase, we can distinguish various learning techniques: (1) Supervised learning (both inputs and outputs are provided to the algorithm), (2) unsupervised learning (only inputs are provided), and (3) reinforcement learning (an objective reinforcement function is maximized). During the inference, the inputs’ data pass through the layers and each layer performs matrix multiplications. The output of the final layer is either a number or a classification output. A deep neural network (DNN) [13] (Figure 2) is an artificial neural network (ANN) with multiple layers between the input and output layers and the operations include linear or nonlinear functions. A special case of DNNs involves the usage of the matrix multiplications with convolutional filter operations, which is common in DNNs that are designed for image and video analysis. This type of models is known as convolutional neural networks (CNNs) [54] and they are used when the numbers of input variables are high. The DNNs designed especially for time series prediction are called recurrent neural networks (RNNs) [55], characterized by having loops in their layer connections to keep state and enable predictions on sequential inputs.

There are many possible choices on how to design a NN model, provided that different hyperparameters of the network bring a different level of accuracy. In particular, a model with high accuracy requires more memory than a model with low accuracy due to the number of parameters. The metric used to measure accuracy depends on the domain in which the ML algorithm is applied. For example, in object detection, the accuracy may be measured by the mean average precision (mAP) [56], which measures how well the predicted object location overlaps with the ground-truth location, averaged across multiple categories of objects.

### 2.2. RNN, GAN, K-NN

A particular type of NN are the RNNs (recurrent neural networks) [57]. In this type of NN, the output values of a high layer are used as input for a lower one. This interconnection allows the use of one of the layers as state memory. Providing a temporal sequence of values as input, it allows us to model also dynamic temporal behavior. This makes them applicable to predictive analysis tasks on data sequences, such as handwriting recognition or speech recognition [58]. A particular RNN is LSTM (long short-term memory) [59]. A LSTM unit is composed of a cell, an input gate, an output gate, and a forget gate. The cell remembers the values over the time and the gates regulate the flow of information into and out of the cell. In particular, the forget gate can learn what information is kept or forgotten during training.

Another type of NN is the generative adversarial network (GAN) [60]. They consist of two networks: Generator and discriminator. The first generates data after it learns the data distribution from a training dataset of real data. The second one is in charge of classifying the real data from the fake ones generated by the generator.

K-nearest neighbors algorithm (K-NN) [61] is an algorithm used in the field of patterns recognition, based on the characteristics of the objects close to the one considered. This method is used both for classification and regression problems. There are different modified versions of k-NN that helps to implement the algorithm in hardware-constrained devices and the most innovative is ProtoNN [62]. It is a k-NN-based algorithm. The main problems of the K-NN for the computation at the edge are: The training data size (the algorithm generates prediction using the entire datasets), the prediction time, and the choice of the distance metric. To address these issues, ProtoNN works on a smaller training dataset excluding the unnecessary data. The dataset is then projected to a low dimension matrix and jointly learned across all data points. Gupta et al. implemented ProtoNN on an Arduino Uno to evaluate its performance using 14 datasets and reported almost the same classification accuracy as the state of the art.

### 2.3. Tree-Based ML Algorithms

Tree-based ML algorithms are used for classification and regression problems that are a very common practice in the IoT field. However, due to the limited resources of the devices, the usual tree algorithms could not be brought on them. An emerging algorithm is Bonsai [63]. The tree algorithm [64] is designed specifically for severely resource-constrained IoT devices and it maintains prediction accuracy while minimizing model size and prediction costs. It learns first a single sparse tree reducing the size model, then it makes nonlinear predictions through the internal nodes and the leaf ones. Eventually, Bonsai learns sparse matrix, projecting all data into a low dimensional space in which the tree is learned. This allows the algorithm to be brought on tiny devices like IoT ones.

The referenced implementation was carried out on an Arduino board populated with an 8-bit ATmega328P microcontroller with 16 MHz operating frequency, 2 kB of Static Random Access Memory (SRAM), and 32 kB of read-only flash memory and on BBC Micro:Bit which has an ARM architecture 32-bit Cortex with an operating frequency of 16 MHz, 16 kB of SRAM and 256 kB of flash.

### 2.4. SVM

**Figure 3:**
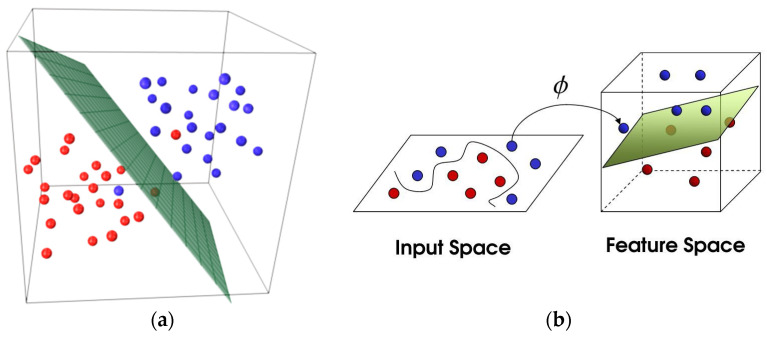
(**a**) Hyperplane that separate two classes of data, (**b**) kernel trick.

One of the most widely used ML algorithms at the embedded level is the SVM [28,29,43,52]. SVM is a supervised learning algorithm that can be used for both classification and regression problems. The algorithm discriminates between two or more classes of data by defining an optimal hyperplane that separates all classes (Figure 3a). The support vectors are the data closest to the hyperplane, which, if removed, would result in a redefinition of the hyperplane itself. For these reasons they are considered the critical elements of the dataset. Usually, the loss function used by the algorithm is the Hinge loss and the optimization function is the descending gradient technique.

Sometimes the data are linearly separable, but this only represents a subset of cases. SVM can efficiently perform a classification using the kernel trick. Suppose we face the problem represented in Figure 3b: It is impossible to find a single line to separate the two classes in the input space. But, after projecting the data into a higher dimension, it is possible to find the hyperplane which classifies the data. Kernel helps to find a hyperplane in the higher dimensional space without increasing the computational cost too much.

## 3. Bringing Machine Learning to the Edge

### 3.1. Architectures

**Figure 4:**
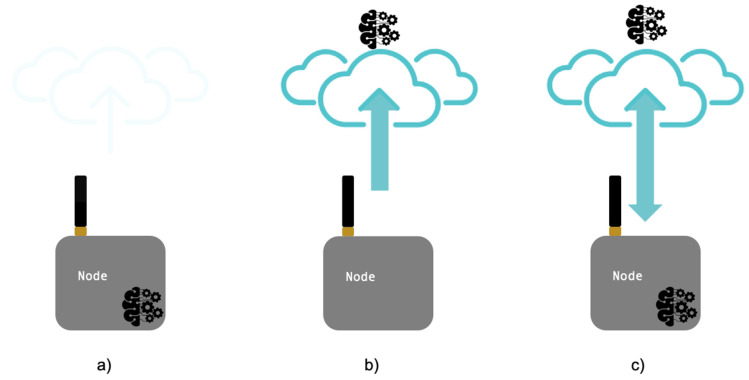
(**a**) On-device computation, (**b**) edge server-based architectures, and (**c**) joint computation.

To meet latency requirements, different architectures for quick-performing model inference have been proposed. The research focused on three important architectures (depicted in Figure 4): (1) On-device computation, where DNNs are executed on the end device; (2) edge server-based architectures (the data are sent from the end devices to edge servers for computation); and (3) joint computation which includes the possibility to have cloud processing.

### 3.2. Model and Hardware

Several research papers focused on the possibility of bringing artificial intelligence to devices with limited resources [44,65,66,67] and there have been efforts in decreasing the model’s inference time on the device. To bring an AI model on embedded devices, ML developers should deal with the proper hardware choice that fits model design and compression.

#### 3.2.1. Model Design

ML developers focus on designing models with a reduced number of parameters in the DNN model, thus reducing memory and execution latency, while aiming to preserve high accuracy. There are several efficient models designed specifically to execute a NN on devices with low computational capacity and power, such as MobileNets [68] or SqueezeNet [69], originally born for computer vision tasks. MobileNets are based on a streamlined architecture that uses depth-wise separable convolutions to build light-weight deep NNs. SqueezeNet downsamples the data using special 1 × 1 convolution filters.

#### 3.2.2. Model Compression

**Figure 5:**
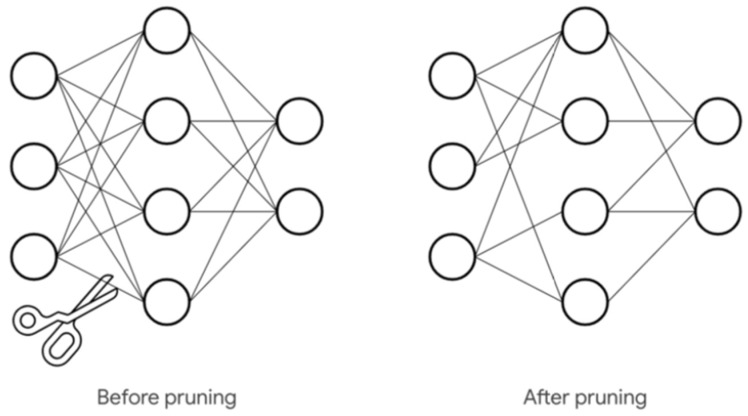
Pruning effect on the network.

The model compression allows us to run the model on tiny devices [70] and there are two main ways to reduce the network: Lower precision (fewer bits per weight) and fewer weights (pruning). Post-training quantization reduces computing power demand and energy consumption at the expense of a slight loss in accuracy. By default, the model weights are float32 type variables, which lead to two problems: Firstly, the model is very large because 4 bytes are associated at each weight, with a considerable memory requirement; secondly, the execution is remarkably slow compared to uint8 type variables. It is possible to considerably reduce the weights from 32 bits to 8 bits, obtaining a 4x reduction in the size of the NN. Note that post-quantization is a technique that is carried out after training the model, but it could be done even before training. ML libraries, such as Tensorflow [71] or Keras [72], give the possibility to apply quantization. As stated above, the reduction of the model size can be obtained not only with quantization, but also with pruning techniques that allow the elimination of connections that are not useful to the NN (Figure 5); this leads to a decrease of the computation request and program memory. Quantization and pruning approaches have been considered individually as well as jointly [70]. These two techniques are the basis of NN compression, from which further techniques have been developed. DeepIoT [73] presents a pruning method for commonly used deep learning structures in IoT devices, and the pruned DNN can be immediately deployed on edge devices without modification. Loss-approximating Taylor expansion was used in [74] as a gradient-based importance metric used for pruning. Anwar et al. [75] selected pruning candidate by hundreds of random evaluations. Yang et al. [76] selected pruning candidate weighted by energy consumption. Early pruning [77] and dynamic pruning [78] explored how to integrate pruning with a better retraining, and saved the retraining time. A technique that is not among those of pruning and quantization, but which has a significant value, is the knowledge distillation. This involves creating a smaller DNN that imitates the behavior of a larger one [79]. This is done by training the smaller DNN using the output predictions produced from the larger one and the smaller DNN approximates the function learned by the larger one.

#### 3.2.3. Hardware Choice

The choice of the algorithm to be used is important to run a model on an edge device. However, this must also be coupled to an optimal choice of hardware. The metric to be used for choosing the hardware is based on accuracy, energy consumption, throughput, and cost [67]. The accuracy of ML algorithms must be measured on a dataset large enough to be able to affirm that the obtained result is valid. Energy efficiency, on the other hand, is closely related to the programmability and size of the NN. By “programmability” we mean the adaptation of the model to the variation of the context, i.e., the model varies the weights as the scenario varies. By “NN size” we mean, instead, the number of layers that the processor must support. The high size and the variability of the scenario imply an increase in terms of computation. In particular, the high size of the NN increases the number of data, and, instead, the programmability involves the need to access the memory, read the weight value, and modify it. This generally involves an increase in energy consumption. By “throughput” we mean the number of operations required in the unit of time and with “cost” the amount of memory required.

Microcontrollers can be used for AI but implementing the algorithm on them is challenging. They are excellent choices in IoT applications and may run networks that are not too large for low-data fusion tasks. A good tool to facilitate the implementation of a DNN on a microcontroller is the X-CUBE-AI [80], suitable only for STMicroelectronics MCUs. It is an expansion of the STM32CubeMX environment that extends the potential of the tool, allowing an automatic conversion of pretrained NNs to low-resource hardware. X-CUBE-AI also optimizes libraries by modifying layers and reducing the number of weights to make the network more “memory-friendly”.

Tiny hardware that can be used for IoT purposes and recommended on the Tensorflow lite website [81] are:

- Arduino Nano 33 BLE Sense [82]
- SparkFun Edge [83]
- STM32 microcontrollers [84]
- Adafruit EdgeBadge [85]
- Espressif ESP32-DevKitC [86]
- Espressif ESP-EYE [87]

Arm has provided its own solution for the IoT field [88]. Recently [89], they announced the introduction of new features on their AI platform, among which new ML Intellectual Property, the Arm^®^ Cortex^®^-M55 processor that can be up to 15 times faster than the previous version, and Arm Ethos™-U55 NPU, the first micro-NPU (neural processing unit) for Cortex-M architecture, which can speed up ML performance by up to 480 times.

A selection of hardware used for IoT devices that implement edge computing is reported in Table 1.

## 4. Edge Server-Based Architectures

The solutions described in the previous sections allow us to run the AI algorithm on end devices but implementing powerful DNNs on tiny devices is still challenging (e.g., decision making and real-time execution). In some circumstances, it is necessary to transfer the computations from end devices to more powerful entities [96]. Since the edge server is close to users, it could be the best approach to solve the problems related to the calculation in optimal times. The easiest way to utilize the edge server is to offload all the computation from end devices to the edge server: The end devices will send their data to a nearby edge server and receive the corresponding results after server processing. When sending data to an edge server, data preprocessing is useful to reduce redundancy and thus decrease communication time. An example could be Glimpse [97] that is a continuous, real-time, object recognition system for camera-equipped mobile devices. Starting from the video, Glimpse identifies objects and labels and traces them. Because the algorithms for object recognition entail significant computation, Glimpse runs almost always on server machines. It uses change detection to filter which camera frames are offloaded. If no changes are detected, Glimpse will perform frame tracking locally on the end device. This preprocessing makes real-time object recognition possible.

Obviously, if the data processing is outsourced to an edge server, more edge devices will belong to it. This should bring the problem of the shared resources. So, the developer should look for the right trade-offs between accuracy, latency, and other performance metrics, such as a number of requests served. A practical solution [98] could be to assign the computation across a hierarchy of edge and cloud servers jointly tuning all the DNN hyperparameters. Mainstream [99] considers a similar scenario but the proposed solution uses transfer learning to reduce the computational resources consumed by each request. Transfer learning enables multiple applications to share the common lower layers of the DNN model and computes higher layers unique to the specific application, thus reducing the overall amount of computation.

## 5. Wireless Standards for AI-Enabled IoT Devices

Most of the energy in the IoT device is wasted due to the communication protocol. Indeed, any unnecessary data that are transferred, stored, and processed appear to be a potential waste of energy. Consequently, excellent algorithms must also be accompanied by efficient communication protocols. According to the specific scenario, the developers can use different communication protocols (Table 2). This is because we can distinguish protocols that allow us to transmit a small amount of data over long distances with a low energy consumption and protocols that can transmit a great amount of data over long distances with a high consumption. If the spectrum band use is considered, we can also classify them into technologies that use the licensed or the unlicensed spectrum, e.g., Industrial, Scientific and Medical (ISM) bands [100]. Among the many communication technologies, we include BLE [101], an example [102] that presents the design and optimization of a smart sensor supplied by 2.4 GHz Radio Frequency (RF) power and performing infrared-based motion detection and BLE communication. Bluetooth wireless technology is widely used, including the introduction of Bluetooth 5 [103,104] that uses less power and supports mesh topology, enables large-scale device networks, and many-to-many communications. Bluetooth 5 meets the requirements for recent IoT devices with its good range, increased speed up to 2 Mbps, and a long-range mode with higher sensitivity at lower bit rates.

ZigBee [105] is one of the main IoT communication standards. It is based on IEEE 802.15.4 [106] standard for WPAN (wireless personal area network) and its primary application is in the field of wireless sensor networks (smart energy and home automation) [107]. It could be used in different fields: Smart cities [108,109], agriculture [110,111], automotive [112,113], and health care [114,115]. ZigBee operates mainly in the 2.4 GHz, but also supports the 868 MHz and 916 MHz ISM bands.

Another solution is Z-Wave, a sub-GHz mesh network protocol often used for security systems, home automation, and lighting controls [116]. Like Zigbee, Z-Wave is a low-power technology based on IEEE 802.15.4 that transfers small amounts of data over short and medium distances. Z-wave uses a proprietary radio system and has a strictly regulated product ecosystem targeting smart homes, while Zigbee devices can be used for a variety of applications and are not fully interoperable.

ANT [117] is a proprietary protocol operating in the 2.4 GHz band designed for low bit rate and low-power networks. It supports point-to-point, star, tree, and mesh networks and up to 65,533 nodes for each of the available channels. It was originally used in sports and fitness sensors but later used for home automation and industrial applications. ANT+ is a standardized layer on top of the ANT protocol allowing devices’ interoperability [118].

In the wake of the market demands of direct IP-based connectivity, new wireless mesh networking standards have been developed. The 6LoWPAN (IPv6 over low-power wireless personal area networks) [119] is a light-weight, IP-based communication and is an open IoT network protocol, primarily used for home and building automation. However, the standard only defines an efficient adaptation layer between the 802.15.4 data link layer and the TCP/IP stack. Thread [120] is a secure and reliable mesh protocol for home automation running over 6LoWPAN and IEEE 802.15.4 radio. The stack is an open standard built as a collection of existing standards and is optimized for low-power operation, but the application layer is not standardized.

The aforementioned WPAN solutions require an application-level gateway that runs the TCP/IP stack via Ethernet or WiFi. Instead, 6LoWPAN-based solutions use an edge router that only forwards packets at the network layer and does not implement an application layer state, allowing low-cost bridging to other IP networks.

WiFi networks (IEEE 802.11) use an access point (AP) as an Internet gateway and have good data capacity and coverage inside buildings. Until recently it was quite expensive to integrate WiFi connectivity into devices with low computing performance, due to the size and complexity of WiFi and TCP/IP software and the high power consumption that make it not suitable for use with battery-powered devices. Now, however, new devices support WiFi and TCP/IP software and have reduced power consumption. The power consumption of these devices can be further reduced by activating the radio section only for short periods, allowing them to operate for over a year with two AA batteries [121].

Many different WiFi protocols are available and operate at either 2.4 GHz or 5 GHz. IEEE 802.11n and IEEE 802.11ac are the most widely used protocols but different versions have been developed in the past years for higher versatility. WiFi HaLow (IEEE 802.11ah) is designed for low data rate and long-range devices [122]. It operates in the sub-GHz ISM band and implements power-saving techniques, such as target wake time (TWT) that wake up the device at defined intervals for a very short time. HaLow was released in 2016, but is not yet widely used in commercial products. The 802.11af [123] has the same target applications of HaLow but it relies on unused TV spectrums in UHF and VHF bands and never took off.

The 802.11ax [124] is a more recent version of WiFi technologies that support higher transfer speed and also introduce power-saving features such as TWT, making it more attractive for IoT applications. It also includes features that allow it to extend the range and allows the partition of the channel into smaller subchannels to reduce the data rates while extending the number of devices that can be reliably connected to an access point, allowing it to scale up to thousands of devices.

Radio-frequency identification (RFID) is a technology that uses sub-GHz ISM bands, designed specifically so devices without batteries could send a signal [125]. NFC (near-field communication) is a protocol used for very close communication [126]. It operates in the 13.56 MHz and is designed to exchange data with another NFC device, allowing bidirectional communications. The low data rate and short communication distance make it suitable only for niche IoT applications.

LoRa (long range) [127,128] is a low-power, wide-area network (LPWAN) technology. It is based on spread spectrum modulation techniques and it could be used for empowering the IoT scenario [129]. In [130], it is presented a solution of machine learning on edge devices with the use of LoRa as a low-power transmission protocol. Implementing machine learning with LoRa allow it to reduce transmitted data by 512 times and extend battery life by 3 times for that specific scenario. Nowadays, the most common strategy for processing data is the use of the cloud, but the transmission of large amounts of data requires frequent recharging of the devices, thus negating the prerogatives of the IoT. In addition, IoT applications could require long-distance data transmission, such as for traffic monitoring. IoT devices must, therefore, have a low-energy profile and sometimes be able to transmit over great distances for a given scenario [125,130,131]. Furthermore, IoT devices require edge processing for bandwidth, latency, and privacy issues. Under these conditions, the efficient use of data reduction and local processing must be coupled with long-range and small-bandwidth transmission protocols and this could be obtained using LoRa.

SigFox [132] is another LPWAN solution. It is a narrowband technology and allows the use of simpler devices that are available from different manufacturers. However, it requires the use of sophisticated and expensive gateways and access points and the network is controlled by SigFox and has a fee. On the contrary, LoRa is open and its use is free with no subscriptions and no constraints on installation of gateways and network servers. However, the production of LoRa radio is a Semtech exclusive.

LTE (long-term evolution), commonly known as “4G LTE”, is a standard for wireless broadband communication based on GSM/EDGE and UMTS/HSPA technologies [133]. After the LTE introduction, it began to compete with the emerging technologies for IoT field such as BLE, narrowband Internet of Things (NB-IoT), ZigBee, and LoRa. The 4G has improved the capabilities of cellular networks but it is not fully optimized for IoT applications [134,135].

NB-IoT [136], has been introduced to provide low-cost, low-power, wide-area cellular connectivity for the Internet of Things. NB-IoT is a standards-based low-power wide-area (LPWA) technology developed to enable a wide range of new IoT devices and services. NB-IoT significantly improves the power consumption of user devices, system capacity, and spectrum efficiency, especially in deep coverage. NB-IoT is built [137] from existing LTE functionalities with essential simplifications and optimizations. At the physical layer, it occupies 180 kHz of spectrum, which is substantially smaller than LTE bandwidths of 1.4–20 MHz. At the higher layers, simplified LTE network functions are supported. Compared to other LPWAN solutions, NB-IoT has the great advantage of eliminating the need for a specific gateway, so sensor data is sent directly to the cloud server, reducing infrastructure costs.

LTE Cat-M1 [138] is a LPWAN that enables cellular services for the IoT world. Compared to NB-IoT, this technology provides higher data rate and the ability to use voice over the network, but requires more bandwidth and, therefore, the devices are more complex and expensive. NB-IoT and Cat-M1 have different and somewhat complementary target applications with the former suitable for small sensors and meters and the latter for devices that require higher data rates and have a higher power budget.

The 5G networks and standards are expected to solve challenges that are facing 4G networks. The 5G is the fifth generation of mobile, cellular technologies, networks, and solutions. Although not just ‘built’ for the Internet of Things (IoT), it will be the major driver of the growth of IoT. The 5G IoT is a novel [139], intelligent network based on 5G communication, which is designed to connect sensing regions (sensors) and processing center (cloud) provided by AI algorithms. It presents the different emerging technologies, involving massive Multiple Input Multiple Output (MIMO) networks, dense static small-cell networks, and mobile small-cell networks. The 5G fulfills the needs of the IoT [135]:

- high data rate;
- high scalable and fine-grained networks, to increase network scalability;
- very low latency;
- long battery lifetime, to support billions of low-power and low-cost IoT devices.

Reducing the latency in the communications, 5G eliminates part of the bottleneck related to the remote execution of ML algorithms [140].

ML developers should properly define the communication technology based on specific design requirements, as well as architectures, hardware, latency, and strategy of computation [141,142,143].

## 6. Joint Computation

**Figure 6:**
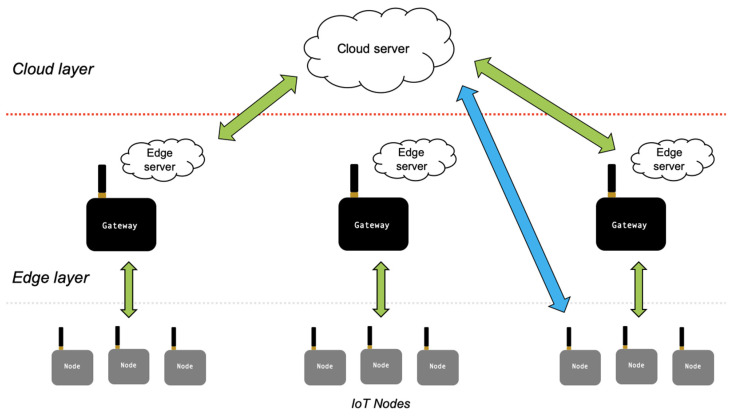
Joint computation among devices, edge, and cloud servers.

Although the edge server can accelerate DNN processing, it is not always necessary to have the edge devices executing DNNs on edge servers. We will introduce three offloading scenarios (Figure 6): (1) partial offloading of partitioned DNN (the decision is what fraction of the DNN computations should be offloaded), (2) hierarchical architectures (offloading is performed across a combination of edge devices, edge servers, and cloud), and (3) distributed computing approaches (the DNN computation is distributed across multiple peer devices).

### 6.1. Partial Offload

In model partitioning approaches, some layers are computed on the device and the others are computed by the edge server or the cloud. This approach can potentially offer latency reductions thanks to the compute cycles of other edge devices. Indeed, after the first few layers of the DNN model have been computed, the size of the intermediate results is relatively small and the output can be sent over the network to an edge server in a faster way than the original raw data [90]. Critical is the choice of the point where the network needs to be partitioned and one algorithm that can be used is Neurosurgeon [173]. It is a light-weight scheduler used to automatically partition DNN computation between mobile devices and datacenters at the granularity of NN layers. So, it decides where to partition the DNN, layer-wise, while accounting for network conditions.

The partition could also be applied to the input data (e.g., raw image) and this is useful for hardware with constrained memory that is largely used in the IoT scenario, such as IoT sensors. However, input-wise partitioning can result in increased data dependence, as computing subsequent DNN layers requires data results from adjacent partitions. DeepThings [90] uses input-wise partitioning.

### 6.2. Hierarchical Architectures

ML algorithm can be performed on edge devices and on the cloud. Entrusting the computational task to the cloud could create a latency problem. Instead, the use of powerful computational cloud resources can potentially decrease the total processing time. For example, Li et al. [45] divided the DNN model into two parts: The edge server computes the initial layers (lower layers) of the DNN model after it received the input data and then the cloud computes the higher layers of the DNN. The cloud sends back the final results to the end devices after processing. In this way, the cloud helps the edge server with the heavier computations. There are also other approaches like DDNN [174] (distributed deep neural networks) in which the computing is distributed across an hierarchical system, consisting of the cloud, the edge (fog), and end devices. DDNN also allows fast and localized inference using shallow portions of the NN at the edge and end devices. Due to the distributed nature, DDNNs improve the fusion of the data from network sensors, system fault tolerance, and privacy for users. Generally, a common feature for the edge approaches is that the edge server serves a limited geographical area, so the input data and, thus, their DNN outputs may be similar.

### 6.3. Distributed Computing

Hierarchical scenario is based on the offload of the network to more powerful entities like edge devices or cloud. In the distributed perspective the DNN computations can be distributed across multiple peer edge devices, like in DeepThings [90]. It distributes the DNN executions between end devices such as Raspberry Pi and Android smartphones. The DNN partition choice is based on the computation capabilities and memory of the end devices.

## 7. Privacy

Both in edge server-based architectures and in joint computation, the data are exchanged over the network (e.g., from end device to edge server or from edge server to cloud) and it may contain sensitive information. This can lead to privacy issues. In fact, as already mentioned, edge servers work locally in a geographically limited area. Therefore, the origin of the data is practically known. Although ML on edge devices allows the data reduction on the network and, therefore, improving privacy by itself, it is possible to improve the system through additional techniques, such as adding noise to data or cryptographic techniques.

### 7.1. Add Noise to Data

A solution is to add noise to the samples uploaded on the network during inference. Wang et al. [175] deployed a smaller DNN locally on the edge device to extract features, add noise to the features, and then upload the features to the cloud for further inference processing by a more powerful DNN. The DNN on the cloud is pretrained with noisy samples so that the noisy inference samples uploaded from the end devices can still be classified with high accuracy at test time. This is based on differential privacy mechanism [176,177,178,179].

### 7.2. Cryptographic Techniques

Cryptographic techniques can be used to compute the inference with a high level of privacy. The target of secure computation [66] is to ensure that the end device receives an inference result without learning anything about the DNN model on the edge server and vice versa. One method of secure computation is homomorphic encryption, in which the communicated data are encrypted and computation can be performed on the encrypted data, as done in CryptoNets [180,181,182]. The DNN is converted in CryptoNets, approximating the common activation functions and operations in a DNN, in a low-degree polynomial, which guarantees the homomorphic encryption. However, a bottleneck of the homomorphic encryption tends to be its compute times. Multiparty computation is another technique for secure computation. In secure multiparty computation, multiple machines work together and communicate in multiple rounds to jointly compute a result. Secure multiparty computation focuses on the privacy of the intermediate computation steps, but its bottleneck tends to be the communication complexity.

## 8. Training

Thus far, edge computing and deep learning have mostly been discussed assuming that a deep learning model has already been trained offline on a prebuilt dataset. This section presents a discussion on training algorithms and hardware for the edge field. Usually, training data produced by end devices would be sent to the cloud, which would then perform the training and finally distribute the trained model back to the edge devices. Leaving data at the edge is useful when privacy is highly desired and also helps to reduce the network bandwidth requirements.

### 8.1. Training Algorithms

Exchanging model parameters and other data between edge devices and cloud servers is mandatory for training an edge–cloud-based DL model. However, as the size of the training model increases, more data needs to be exchanged between edge devices and servers. The high network communication cost is a bottleneck for a training model, and a local edge training implementation is required. An example of local networks is a mobile computing system for DNN applications (MoDNN). MoDNN [183] uses a pretrained DNN model and scans each layer of the DNN model to identify layer types. If the layer is a convolutional one, the input layer is divided by biased one-dimensional partition (BODP) method. BODP decreases the computing by reducing the input size. If a fully connected layer is detected, the layer input is assigned to different work nodes (mobile devices) to achieve the minimum total execution time. In this case the network does not change the weights as the external scenario varies because it is pretrained. However, the structure of the edge system is hierarchical and the training can be distributed among peer edge devices and the cloud. In-edge AI [6] is a framework which allows better collaboration among devices and edge nodes to exchange the learning parameters for a better training and inference of the model. It integrates deep reinforcement learning techniques and federated learning for mobile computing purpose. Teerapittayanon et al. [174] used a cloud server for training the DDNN among different devices (including edge devices and the cloud), while the most powerful one trains the network. The training of DDNNs is difficult because of multiple exit points. To address this issue, the network was trained jointly by combining losses from each exit point during back-propagation. The training could be made also on pruned model: Chandakkar et al. [184] designed a new architecture to retrain a pruned network on an edge device. A complete DNN is trained for an epoch (when an entire dataset is passed forward and backward through the DNN) on the original data. Also, layer-wise, magnitude-based weight pruning is performed with a user-defined threshold value. This approach greatly reduces the computational complexity by removing connections in a DNN model and makes it suitable to run on a limited resources device. Unfortunately, any pruning process reduces the accuracy of a model. To overcome this issue, this approach finds the indices of the most important weights for an important feature and excludes these elements from being pruned. Finally, the pruned DNN network is used while training the next epoch because these operations are performed cyclically.

To reduce communication costs and keep model accuracy high, Tao and Li introduced a new method called edge stochastic gradient descent (eSGD) [185]. In this approach, all edge devices run training tasks separately with independent data and the gradient values generated by the edge devices are sent to the cloud servers. The server, after obtaining the gradients from the end devices, uniforms the gradients by performing the average. After that, it updates the parameters by using this average value. These updated parameters are sent back to the edge devices for the next training step. This process is called parameter synchronization. Unfortunately, this gradient selection technique decreases model accuracy. The eSGD uses two mechanisms to maintain a high level of accuracy for the training:

- ‘Important’ updating: After each mini-batch, only a small fraction of the gradient coordinates need to be updated. The algorithm determines main gradients, which will then be updated by the server. This process significantly reduces communication cost.
- Momentum residual accumulation: This mechanism is applied for tracking and accumulating out-of-date residual gradients, which helps to avoid low convergence rate caused by the previous important updating method.

The eSGD is capable of reducing the gradient size of a CNN model by up to 90%. Unfortunately, high gradient shrinking leads to bad accuracy. Tao and Li used Modified National Institute of Standards and Technology (MNIST) database in their experiments and reported 91.22% accuracy with a 50% gradient drop.

### 8.2. Training Hardware

Updating the neural network or computing complex algorithms cannot be completely entrusted to tiny hardware like microcontrollers. Field-programmable gate array (FPGA) and graphical processing unit (GPU) consume too much power (the FPGA is still a better choice than the GPU since it is versatile and consumes less power), but they turn out to be excellent for training NNs or performing powerful algorithms [186]. GPUs use temporal architectures such as SIMD (single instruction multiple data) or SIMT (single instruction multiple threads) to perform the MACs in parallel and there are software libraries designed for GPUs that optimize the matrix multiplications e.g., NVIDIA CUDA® Basic Linear Algebra Subprograms (cuBLAS) [187], NVIDIA CUDA® Deep Neural Network library (cuDNN) [188]. The matrix multiplications on these platforms can be further improved by applying transforms to the data to reduce the number of multiplications. Fast Fourier transform (FFT) [189] is a well-known approach that reduces the number of multiplications from *O((N^2^_o_)(N^2^_f_))* to *O*(*N^2^olog*_2_*No*), where the output size is No*No and the filter size is *Nf* *Nf; however, the benefits of FFTs decrease with filter size. Other approaches include Strassen [190] and Winograd [191].

Recently, some very interesting devices are emerging, such as the Hailo-8 DL [192] from the Hailo company. The Hailo-8 DL is a processor suitable for performing deep-learning at high levels and allows for very high performance on end devices with minimum power consumption, size, and costs. In particular, it offers high performance (26 tera-operations per second) and is very efficient and highly flexible (reprogrammable). Google has developed an ASIC (application-specific integrated circuit) dedicated to the TensorFlow library TPU (Tensor processing unit) [193], whose computational capacity is 180 teraflops. These are examples of IA accelerators, such as NPU, that is a class of microprocessors designed to provide hardware acceleration to artificial NNs, automatic vision, and ML algorithms for robotics, IoT, and other data-based applications [194]. While hardware DNN accelerators are quite new, there have already been two branches of designs. The first class of accelerators only looked at the data flow, ignoring the memory energy consumption. The second one tried to address the amount of energy consumption due to memory access. The first style of accelerators include ConvNet Processor (CNP) [195], Neuflow [196], and dynamically configurable (DC) CNN [197], proposing customized logic to map convolution to hardware with more parallelism and flexibility. The second wave of accelerators focused on optimizing memory transfer and data movement. As modern NNs get larger, researchers realize that memory access and moving data is more critical than matrix products between layers. Among these accelerators (TPU is included in this class of accelerators), DianNao [198] implements an array of multiply-add units to map large DNNs onto its core architecture. It has customized on-chip buffer to minimize Dynamic Random Access Memory (DRAM) traffic. DaDianNao [199] and ShiDianNao [200] eliminate the DRAM access by having all weights on-chip. An interesting AI accelerator is Movidius stick [201] suited for edge computing because it makes easy to add deep learning capabilities to existing computing platforms. It is designed mainly for computer vision tasks at the edge [202] and allows deploying CNNs on low-power applications that require real-time inferencing. A detailed guide on the use is reported in [203].

Another AI accelerator is Coral [204]. It is a platform from Google that allows realizing devices with local AI, providing hardware acceleration for neural networks at the edge of the network without any help from the clouds. At the base of Coral there is Google’s Edge TPU, an ASIC chip optimized to run lightweight machine learning algorithms. Many applications are reported by Coral project itself [205]. A selection of AI accelerator devices that implement edge computing is reported in Table 3.

## 9. MNIST Example

In this section we will analyze how it is possible to port a NN to the embedded environment using one of the most famous models in machine learning world: the MNIST, the ML “Hello World”. We will show how to create the neural network using the Tensorflow library and subsequently Keras, using techniques such as pruning and quantization to reduce the size of the model. Finally, the algorithm will be implemented on the NUCLEO-F746ZG board through the X-CUBE-AI tool.

### 9.1. Dataset

The dataset MNIST was developed by Yann LeCun [216], Director of the Facebook Research Center for Artificial Intelligence, to recognize numeric digits. The dataset was created from a series of documents made available by the NIST (National Institute of Standards and Technology) [217] and the images were normalized in size and centered. In particular, the dataset provides 28 × 28 handwritten images with a total of 784 pixels per image, with a splitting of the dataset to implement training and evaluation of the model to overcome the overfitting issue. The training set consists of 60,000 samples and the test set of 10,000 samples. The objective is to write an algorithm that allows recognizing which digit has been written. Since there are 10 types of digits (numbers from 0 to 9), the problem can be seen as clustering task with 10 possible classes. In a first instance, we will show the realization of the NN using a DNN with 2 hidden layers between input and output; then we will show how to implement the same problem using a CNN and Dropout [218] to increase the accuracy of the model.

### 9.2. Model with Tensorflow

In this first investigation, we will present the implementation of the NN with Tensorflow. The image was saved in a vector of 784 elements in which each element corresponds to the intensity of the color associated to the pixel. With the samples normalized, the values closer to 0 are close to white, and those closer to 1 are classified as black. As already said, this is a classification problem, so the targets are categories. One way to represent the classes is one hot encoding, which is optimal in the case of limited classes: The target for each sample fed to the NN is a vector of length 10 (e.g., if we feed the NN with the digit of value 4, the target associated with it should be [0,0,0,0,1,0,0,0,0,0]).

The NN consists of an input layer (dimensions: 784), two hidden layers (dimensions: 50), and an output layer (dimensions: 10). Since we are working with a DNN, activation functions are mandatory. On the basis of several test carried out, the choice falls on a *relu* for the first layer and a *sigmoid* function for the second layer, as this couple produce the higher level of accuracy (Table 4).

The loss function used, being a classification problem, is the cross entropy applied directly on the *softmax*. The function *tf.nn.softmax_cross_entropy_with_logits(logits, labels)*, combines the two operations making it faster, but also numerically stable. Instead, as optimization function it is possible to use an adaptive function, i.e., ADAM. Once the model is defined, we have all the requirements for the training of the NN. Before starting the training, it is necessary to initialize the variables (weights and biases) using the *tf.global_variables_initializer()* method. Tensorflow uses Xavier [219] as default initialization. Then, it is necessary to define the size of the batches, their number as a function of the size of the dataset, and a threshold for the loss function related to the validation dataset, so that if the error increases, early stopping prevents overfitting. It should be noted that the threshold has been set at a high value, so that early stopping will not occur at the first time. The final part of the required code is related to the actual training, realized through a cycle. For each period, which is repeated to be a complete iteration of the dataset, a relative to the batches is defined, through which it is possible to calculate the average error relative to the single period as the sum of the errors associated with the batches on the number of batches. At the end of each epoch, it is possible to calculate the loss relative to the validation dataset; if the current validation error is higher than the previous one, then the model has conformed too much to the dataset and, therefore, has no ability to adapt and it is necessary to stop learning using early stopping.

The model made with Tensorflow is too heavy in terms of memory occupation for an edge application; in this example the NN weighed around 15 MB. Tensorflow Lite (TFLite) [220] was created specifically to overcome this problem, proposing a set of tools that help programmers to run embedded, mobile, and IoT devices IA models. In the following, we will show how to use TFLite to bring the NN on a microcontroller. The workflow that we will follow in this tutorial is the following:

- Definition of the model in Keras (using Tensorflow backend),
- Conversion of the model from Keras to TFLite,
- Implementation of a post-training quantization to further decrease the dimension of the NN,
- Design of a Graphical User Interface (GUI) to draw the digit, and
- Test on hardware devices.

### 9.3. Keras Model

The NN can be built using CNN and the dropout technique. The use of CNN is not necessary, but it is recommended since the number of input variables is very high (CNN allows training the model on a smaller dataset, reducing considerably the number of parameters to learn). The model is defined using the *Sequential()* method which, according to the documentation [221], allows defining the model as a linear stack of layers. The first layer is a 2D convolution layer that creates a convolutional kernel that is superimposed convolutionally with the input layer to produce an output tensor.

To the second layer, also convolutional, is added the *MaxPooling* operation for spatial data (2D). The Dropout is then applied to the network in input. The last two layers are densely connected layers; at the penultimate layer a *relu* is applied as activation function, whereas at the last one a *softmax*. Finally, for the training phase, *crossentropy* is used as loss function and *Adadelta* as optimization function. A summary of the parameters is shown in Table 5.

After defining the model and parameters for the training phase, it is possible to train the network. With the presented configuration, it is possible to observe that the use of a CNN is able to increase the model accuracy from 96% to 99%.

### 9.4. Tensorflow Lite

The model in Keras was too heavy for an embedded solution (7172 kB), having embedded devices memories of a few hundred of kB. However, TFlite and the TFliteConverter tool allows considerably scaling the NN down to 2.4 MB. TFlite is characterized by two main components [222]:

- The interpreter runs the optimized models on different hardware types (including mobile phones, low computational capacity devices, and microcontrollers), and
- The converter, which converts the model to a more efficient format for use by the interpreter.

In our case, the converter was used to adapt the model to the TFLite format (serial format is based on FlatBuffers library [223]).

model = ‘Model_Keras_MNIST_CNN_Test.h5’.
converter = tf.lite.TFLiteConverter.from_keras_model_file(model)
tflite_model = converter.convert()

Tensorflow provides tools (Tensorflow Model Optimization Toolkit) for the optimization of the model. The toolkits support techniques used to:

- Reduce latency and inference costs, and
- Implement IA models on edge devices with limited capacity and low-power profile.

These techniques include post-training quantization and pruning techniques. Unfortunately, the quantization of TFLite models is not supported by X-CUBE-AI and, therefore, we selected the NN compression adopted by the ST software.

### 9.5. Pruning

The reduction of the model can be obtained not only with quantization techniques, but also with pruning techniques that allow eliminating connections not essential for the NN and consequently reduce the number of computations and the demand of memory space for the NN. Also, for this purpose, it is possible to use the libraries provided by TensorFlow and their examples [224]. As discussed in the previous paragraph, the network is redefined importing the *tensor-flow_model_optimization* Application Programming Interfaces (APIs). The APIs can be applied either to the single layer or to the whole model. In our example, we applied the APIs to the single layer. The pruning technique consists of iteratively removing connections between layers, given a sparsity parameter (percentage of weights eliminated) and scheduling (pruning frequency). To help the model convergence, connections should not be eliminated immediately but every tot; in this example we set the elimination starting from 2000 step every 100 steps. Next, it is necessary to define, among the pruning parameters, the end step. Then, the model is defined by setting the pruning parameters and applying them to the NN. Finally, it is possible to convert the model and make the quantization. The technique reduces the number of parameters and the computations, preserving the model’s accuracy in terms of predictions. The main impact is due to quantization, but also pruning contributes to this purpose by increasing the inference speed and reducing the amount of energy used, thus allowing the use of the IA model on devices with low energy profile and low computational power.

### 9.6. Graphical User Interface

**Figure 7:**
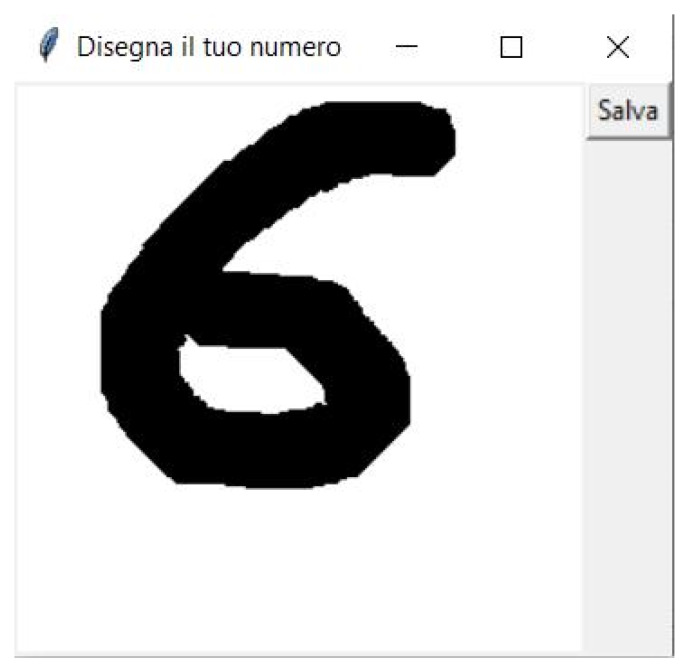
Digit “6” drawn by the user.

To test the model validity once brought to the microcontroller, a suitable GUI that allows the user to type the numeric digit can be used, as well as the direct transfer of saved handwritten digit as input data. The GUI can be made with PIL [225] (Python Imaging Library), Tkinter [226], de facto standard of GUIs in Python. The GUI allows drawing the numeric digit using the mouse motion on the canvas object (Figure 7). Once the drawing is finished, it is possible to export the image as csv, a format supported for the validation on target made by X-CUBE-AI tool.

### 9.7. Validation on Target

**Figure 8:**
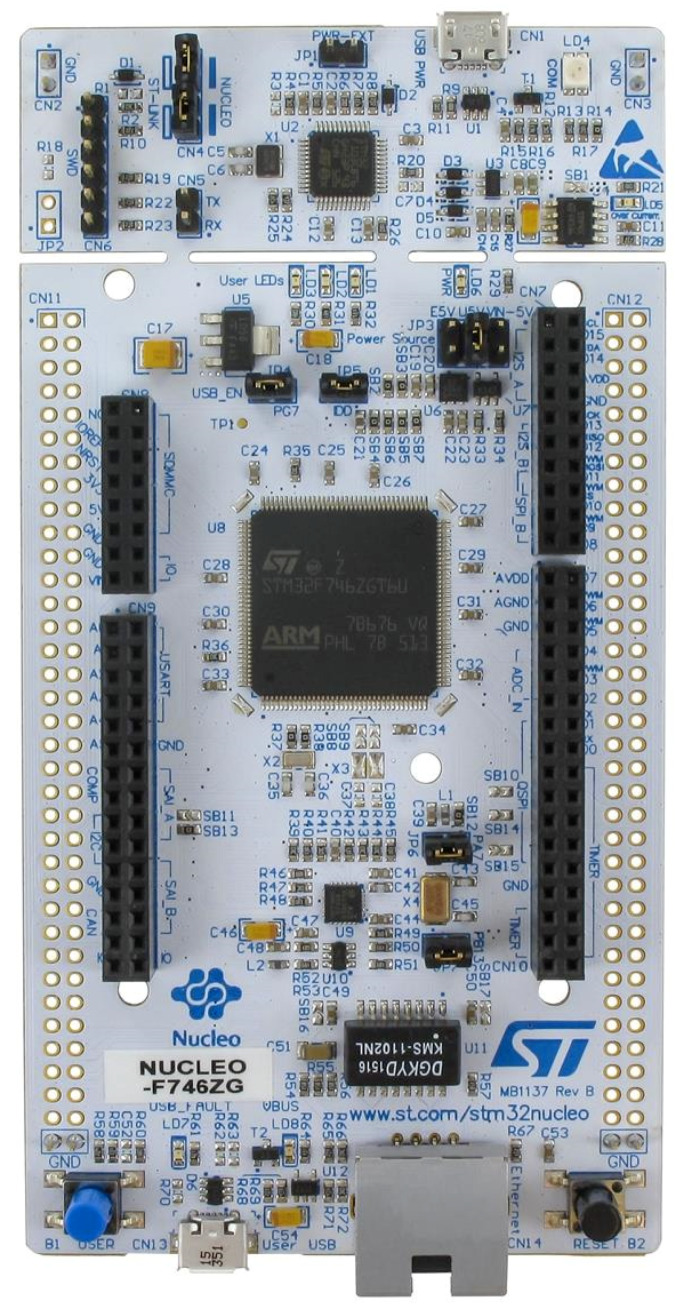
STMicrolectronics NUCLEO-F746ZG.

The STMicroelectronics NUCLEO-F746ZG board [227] (Figure 8) and the STM32CubeMx tool were used for deploying the model to a real hardware. To guarantee higher performance, the operating frequency of the microcontroller must be set to 216 MHz, and the cache should be enabled. To test the model on the microcontroller, it is necessary to enable the X-CUBE-AI tool by choosing *Validation* as project mode. The Universal Synchronous-Asynchronous Receiver/Transmitter (USART) can be used to let the Personal Computer (PC) communicate with the microcontroller. The artificial intelligence tool [80] is used to bring the model on the device. The tool does not yet support techniques such as quantization for NNs defined with TFlite and pruning, but it is possible to load on the microcontroller the nonquantized model using the compression provided by the program itself. In particular, the compression method aims to optimize memory usage both in terms of Read-only memory (ROM) and RAM, using a dataset-less approach. The reduction of the NN by the tool is made possible through the use of various expedients [80]:

- Weight compression: It is applicable only to dense layers (or fully connected layers) and is based on weight-sharing algorithms such as K-means clustering.
- Layers fusion: It allows merging two layers to optimize data placement, decreasing the number of the DNNs layers (e.g., nonlinearities or pooling after a convolutional layers).
- Activation function optimization: Part of the memory is used to store temporary hidden layers values, so activation memory is reused across different layers.
- Once the model is compressed (in this example we opted for a x4 compression), the tool gives the possibility to make an analysis of the NN to understand if it is loadable on the chosen microcontroller and to visualize the diagram of the loaded model. The Table 6 reports the output analysis of the network implemented in the example. It includes:
- RAM: Indicates the size of the memory required to store the intermediate calculations;
- ROM/Flash: Indicates the memory size needed to store weight and bias after compression; and
- Complexity: Reports the complexity of the model in MAC (multiply-accumulate operations), unit of measure used also to express the complexity of the activation functions.

Finally, it is possible to proceed to the validation to compare the defined model with the one generated in C language by the ST tool, feeding both models the same set of data. The validation can be carried out as:

- Validation on desktop: The model in C is executed on the PC.
- Validation on target: The generated model is executed on the device of interest. It is necessary to load the code on the microcontroller and set a serial communication to communicate with the host.

**Figure 9:**
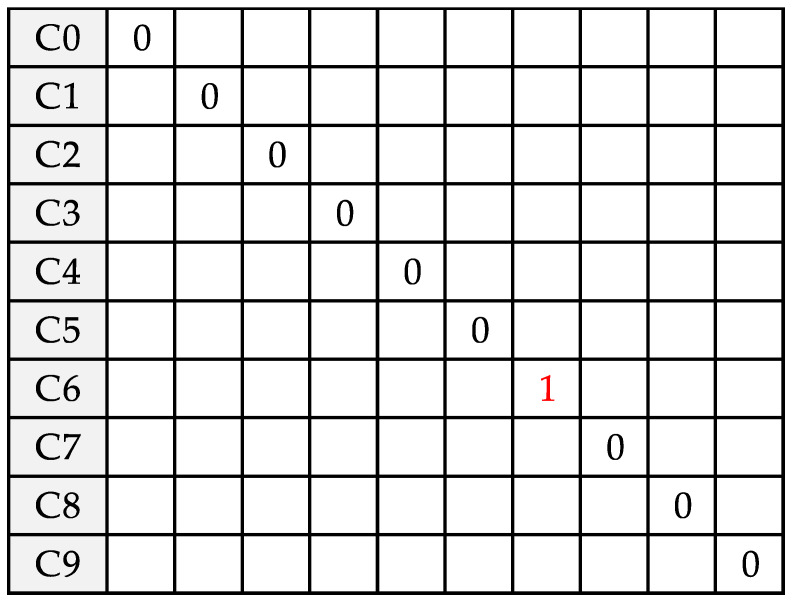
Inference result showing the recognition of the digit “6” drawn by the user (accuracy = 100.00%, root-mean-square error (rmse) = 0.0000, medium average error (mae) = 0.0000, 10 classes, 1 sample).

**Figure 10:**
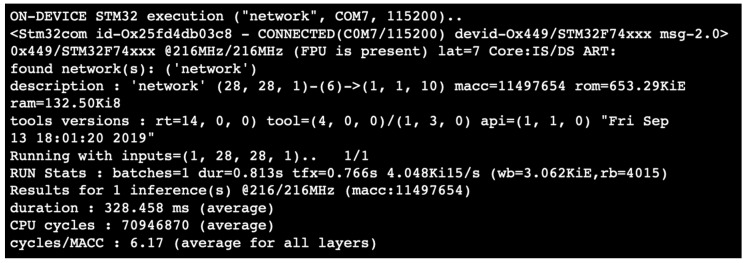
Inference details.

In both cases the data can be either randomly generated by the tool or can be imported from outside as csv file. After loading the code on the microcontroller, it is possible to enable the validation on the target, and to load the data generated using the GUI as input. The STM32CubeMx reports the model results and it is possible to notice that, in this case, the NN allows effectively recognizing the numeric digit between 10 classes (Figure 9) with an accuracy of 100.00%, root-mean-square error (rmse) = 0.0000, and medium average error (mae) = 0.0000. In Figure 10, the results obtained during validation are reported; the calculation took about 330 ms and the execution time layer by layer is shown in Table 7.

With this example, the full implementation of an eML application of image recognition has been designed and put into practice with good performances.

## 10. Conclusions

Deploying machine learning on Internet of Things devices reduces the network congestion by allowing computations to be performed close to the data sources, preserving privacy in uploading data, and reducing power consumption for continuous wireless transmission to gateways or cloud servers. The aim of this work was to provide a review of the main techniques that guarantee the execution of machine learning models on hardware with low performances in the Internet of Things paradigm, paving the way to the *Internet of Conscious Things*.

In this work, a detailed review on models, architectures, and requirements on solutions that implement edge machine learning on IoT devices was presented, with the main goal to define the state of the art and envisioning development requirements.

The review focused on ML systems deployed on edge devices, providing a comparison between the ML algorithms implementable in edge computing. In addition, the process of bringing ML to the edge was analyzed in detail, considering edge server-based architectures and joint computation, thus envisioning both the case of the absence (and the related effect on *privacy* and *local computational* operations) and the presence (and how it impacts on *cloud/edge server* communications and remote *data transmission* power consumption) of data transmission to gateways or servers.

The actual state of development of edge computing foresees a series of variegated solutions able to satisfy a plurality of needs. Depending on the requirements (privacy, energy consumption, computational complexity), it is possible to define a set of compatible hardware and software to implement AI-enabled IoT effective solutions.

An example of edge machine learning implementation is provided in the review, demonstrating the effectiveness and ease of use of the proper edge-platform used for implementing the machine learning “Hello World”.

## Figures and Tables

**Table 1:** Hardware used for Internet of Things (IoT) devices that implement edge computing.

Work	DNN Model	Application	End Devices	Key Metrics
This work (Section 9)	CNN	Image Recognition	STM32F401RE (ARM^®^ Cortex^®^ -M4)	fast inference
[23]	SVM	Image Recognition	Raspberry Pi model 3 (ARM^®^ v8)	fast inference
[90]	DNN	Distributed Computing	Raspberry Pi model 3 (ARM^®^ v8)	hierarchical
[91]	SVM, CNN	Video Analysis	Raspberry Pi model 3 (ARM^®^ v8)	fast inference
[92]	SVM	Video Analysis	Raspberry Pi model 3 (ARM^®^ v8)	fast inference
[28]	SVM	Battery Lifetime Estimation	SPHERE	energy
[44]	CNN	Image Recognition, Sensor Fusion	Motorola 68HC11	fast inference
[65]	SVM	Code execution	ARM^®^ v7	accuracy
[93,94]	Logistic Regression	Human Activity Recognition	ESP32	accuracy
[95]	CNN	Speech Recognition	Sparkfun Edge	accuracy

**Table 2:** Main communication technologies used in IoT.

Group	Technology	Data Rate	Distance (Indoor/Outdoor)	Works
Contactless	NFC	424 kbps	0–4 cm	[126]
Contactless	RFID	640 kbps	10–20 m	[125]
LPWAN	LoRa	0.3 to 50 kbps	5–10 km	[127,128,144,145,146,147,148]
LPWAN	SigFox	100 or 600 bps	30–50km	[143,148,149,150,151]
WPAN	Zigbee	250 kbps	10–100 m	[152,153,154,155]
WPAN	Z-Wave	100 kbps	100 m	[116,156]
WPAN	Bluetooth LE	1 Mbps	10 m/50 m	[102,157,158,159]
WPAN	Bluetooth 5	2 Mbps	40 m/200 m	[160,161,162]
WPAN	ANT	60 kbps	30 m	[163]
WiFi	IEEE 802.11n	600 Mbps	70 m/250 m	[164]
WiFi	IEEE 802.11ax	9600 Mbps	30 m/120 m	[124]
WiFi	IEEE 802.11af	570 Mbps	280 m/1 km	[165,166]
WiFi	IEEE 802.11ah	347 Mbps	140 m/500 m	[122,166,167]
Cellular	NB-IoT	200 kbps	280 m/1 km	[136,137,150,168]
Cellular	LTE-M1	1 Mbps	5–100 km	[138]
Cellular	4G/LTE	150 Mbps	15 km	[169]
Cellular	5G	10–50 Gbps	2 km	[170,171,172]

**Table 3:** Artificial Intelligence (AI) accelerator devices that implement edge computing.

Work	DNN Model	Application	End Devices
[206,207,208]	SVM/CNN	Image and Video Analysis	Movidius
[209,210,211]	CNN	Image and Video Analysis, Robotics	Jetson TX1
[212,213]	YOLO [214]	Image Recognition, Robotics	Jetson TX2
[98]	AlexNet	Image Classification	Nvidia Tegra K1
[196]	CNN	Image Analysis	Neuflow
[215]	CNN, DNN	Image Recognition	DianNao
[200]	CNN	Vision Processing	ShiDianNao

**Table 4:** Accuracy for different activation functions.

First Level	Second Level	Accuracy on Test
relu	relu	96.20%
tanh	tanh	96.80%
sigmoid	sigmoid	96.96%
relu	tanh	97.18%
tanh	relu	96.64%
sigmoid	relu	96.88%
relu	sigmoid	97.25%
tanh	sigmoid	97.21%
sigmoid	tanh	97.10%

**Table 5:** Model outline.

Layer (Type)	Output Shape	Param #
conv2d_1 (Conv2D)	(None, 26, 26, 32)	320
conv2d_2 (Conv2D)	(None, 24, 24, 64)	18496
max_pooling2d_1 (MaxPooling2)	(None, 12, 12, 64)	0
dropout_1 (Dropout)	(None, 12, 12, 64)	0
flatten_1 (Flatten)	(None, 9216)	0
dense_l (Dense)	(None, 64)	589888
dropout_2 (Dropout)	(None, 64)	0
dense_2 (Dense)	(None, 10)	650

#: Total parameters, 609,354; trainable parameters, 609,354; nontrainable parameters, 0.

**Table 6:** Prediction of model on hardware.

Name	RAM	FLASH	Complexity
Network	135.68 kBytes	668.97 kBytes	11497654 MAC

**Table 7:** Time contribution of each layer.

Description	Shape	ms
10004/(2D Convolutional)	(26, 26, 32)	9.328
10011/(Merged Conv2d/Pool)	(12, 12, 64)	299.524
10005/(Dense)	(1, 1, 64)	19.562
10009/(Nonlinearity)	(1, 1, 64)	0.006
10005/(Dense)	(1, 1, 10)	0.022
10009/(Nonlinearity)	(1, 1, 10)	0.014
		328.458 (total)
